# Hydration of Proton-conducting BaCe_0.9_Y_0.1_O_3−δ_ by Decoupled Mass Transport

**DOI:** 10.1038/s41598-017-00595-w

**Published:** 2017-03-28

**Authors:** Dae-Kwang Lim, Ha-Ni Im, Sun-Ju Song, Han-Ill Yoo

**Affiliations:** 1 Department of Materials Science and Engineering, Chonnam National University, Gwangju, 61186 Republic of Korea; 2 Department of Materials Science and Engineering, Seoul National University, Seoul, 08826 Republic of Korea

## Abstract

Mass relaxation profile of a perovskite-type oxide, BaCe_0.9_Y_0.1_O_3−δ_, was studied to understand decoupled diffusion of oxygen and hydrogen species during hydration/dehydration. The mass relaxation measurements are performed by thermogravimetric analysis (TGA) under various humidity conditions (Dry, −3.0 ≤ log(*p*H_2_O/atm) ≤ −1.6) at a constant oxygen partial pressure (log(*p*O_2_/atm) = −1.00 ± 0.01). The decoupled ions participated in hydration/dehydration reactions were proven to be at different ratios from the result introduced by the 8*R*_*m*_ function. The enthalpy and entropy of non-stoichiometric hydration reaction, which considers each ratio of charge-carrier species, were −144.7 ± 3.7 kJ/mol and −147.8 ± 3.2 J/mol · K, respectively.

## Introduction

Among many well-known perovskite proton-conducting materials, acceptor-doped barium cerate and barium zirconate (BaCe_1−x_M_x_O_3−δ_, BaZr_1−x_M_x_O_3−δ_; M = Y, Yb) have been the most studied materials for application in practical solid-state devices, because they have high proton conductivities at intermediate temperatures (500–800 °C)^1–5^. The proton concentration^6–8^ and chemical diffusivity of water^9–11^ in these materials, as functions of thermodynamic parameters, have been investigated for their specimens exposed to sudden changes in water vapor content because of the functionality as solid electrolyte. However, in these proton conductors some recent reports, such as the electrical conductivity relaxation measurements showing a non-monotonic two-fold relaxation behavior^12–14^ and *in situ* optical absorption spectroscopy with impedance spectroscopy^15^, have demonstrated that in the p-type oxidizing regime the proton transport depends on the two decoupled ambipolar diffusivities of proton and hole ($O{H}_{O}^{\cdot }$-${h}^{\cdot }$) and (${V}_{O}^{\cdot \cdot }$-$2{h}^{\cdot }$) pairs. Accordingly, the decoupled mass- and charge-transport theory, which was earlier based on the chemical diffusion of water, has been successfully revised by Yoo *et al*.^16^.

The objective of this work is to prove the non-stoichiometric water diffusion by studying mass relaxation during hydration/dehydration process involving the decoupled diffusion via thermogravimetric analysis (TGA) for 10-mol% Y-doped BaCeO_3_ materials under various thermodynamic conditions. The mass relaxation experiments using TGA were uniquely performed to clearly confirm the reaction of decoupled diffusion of hydrogen and oxygen upon hydration and dehydration in a p-type conductor. The weight change experiments also allowed the calculation of the equilibrium constant of the overall nonstoichiometric water uptake reaction as the ratio of charge carrier concentrations based on the concentrations of decoupled hydrogen and oxygen.

## Theoretical background

In the mass-relaxation, the chemical diffusion fluxes *J*_*i*_ and *J*_*v*_, from Supplementary equations (S5) and (S6), are oriented parallelly during hydration or dehydration. The overall weight change of the specimen can be represented as:

1 $$(m-{m}_{0})/{V}_{0}={{\rm{w}}}_{i}({\bar{C}}_{i}-{C}_{i,0})+{{\rm{w}}}_{v}({\bar{C}}_{v}-{C}_{v,0})$$

where *m*_0_ is the initial specimen-mass at *t* = 0, and w_*i*_ and w_*v*_ are the mass per unit concentration of protons and oxygen, respectively. Although the volume of sample (*V*_0_) may change under hydration or dehydration, it is assumed to remain constant because the overall difference may be negligible. The values for w_*i*_ and w_*v*_ are the molar weight of the carrier species, which are defined as 1 g/mol and 16 g/mol, respectively when protons and oxygen ions are solely responsible for the mass-transfer.

The closed-form solution for hydration or dehydration is ref. 13:

2 $${\rm{\Delta }}m=m-{m}_{0}=({B}_{i}+{B}_{v})-{B}_{i}f({\tau }_{i})-{B}_{v}f({\tau }_{v})$$

with

3 $${B}_{i}\equiv {{\rm{w}}}_{i}{V}_{0}({C}_{i,\infty }-{C}_{i,0})$$

4 $${B}_{v}\equiv {{\rm{w}}}_{v}{V}_{0}({C}_{v,\infty }-{C}_{v,0})$$

The range of concentration of *k* = *i*, *v* is ${C}_{k,0}\le {\bar{C}}_{k}\le {C}_{k,\infty }$ for hydration and ${C}_{k,0}\ge {\bar{C}}_{k}\ge {C}_{k,\infty }$ for dehydration.

The external equilibria are expressed, including the equilibrium constant of the hydration reaction in Supplementary equation (S3), by the following equations^17, 18^:

5 $${K}_{1};{H}_{2}O+{V}_{O}^{\cdot \cdot }+{O}_{O}^{\times }\leftrightarrow 2O{H}_{O}^{\cdot }$$

6 $${K}_{2};{H}_{2}O+2{h}^{\cdot }+2{O}_{O}^{\times }\leftrightarrow 2O{H}_{O}^{\cdot }+\frac{1}{2}{O}_{2}(g)$$

7 $${K}_{3};\frac{1}{2}{O}_{2}(g)+{V}_{O}^{\cdot \cdot }\leftrightarrow {O}_{O}^{\times }+2{h}^{\cdot }$$

The corresponding equilibrium constants are given by:

8 $${K}_{1}=\frac{{[O{H}_{O}^{\cdot }]}^{2}}{[{V}_{O}^{\cdot \cdot }]\cdot [{O}_{O}^{\times }]\cdot p{H}_{2}O}$$

9 $${K}_{2}=\frac{{[O{H}_{O}^{\cdot }]}^{2}\cdot p{O}_{2}^{1/2}}{{p}^{2}\cdot p{H}_{2}O\cdot {[{O}_{O}^{\times }]}^{2}}$$

10 $${K}_{3}=\frac{{p}^{2}\cdot [{O}_{O}^{\times }]}{p{O}_{2}^{1/2}\cdot [{V}_{O}^{\cdot \cdot }]}$$

The relationship of the equilibrium constants *K*_1_, *K*_2_, and *K*_3_ can be expressed as:

11 $${K}_{1}={K}_{2}\cdot {K}_{3}$$

Defining a quantity *R*_*m*_ as the reaction of the incorporation of hydrogen and oxygen for the hydration of the sample, or the reaction of the separation of these carriers for dehydration, the *R*_*m*_ for mass relaxation upon water uptake can be derived from equations (3) and (4) as ref. 13:

12 $${R}_{m}=\frac{{B}_{i}}{{B}_{v}}=\frac{{w}_{i}{\rm{\Delta }}{c}_{i}}{{w}_{v}{\rm{\Delta }}{c}_{v}}$$

*R*_*m*_ has a value of 1/8 when the reaction of water occurs by *K*_1_ (equation (5)), which is directly proportional to H_2_O. In the uptake of decoupled hydrogen and oxygen, the ratio of excess oxygen in the reaction (*x*) is denoted as:

13 $$x=\frac{1}{8{R}_{m}}$$

where 8*R*_*m*_ is the ratio of the changes in the concentrations of hydrogen versus oxygen for a hydration reaction; it is equal to 1 when incorporating H_2_O. The value of *x* has three distinguishable regimes; 1) *x* = 1 occurs with water, 2) *x* < 1 occurs for an excess of protons, and 3) *x* > 1 occurs for an excess of oxygen ions. The ratio of the concentrations of hydrogen and oxygen ions can be calculated with the parameter *x* defined by the *K*_1_ and *K*_2_ (equation (6)) reactions. Therefore, the weight of the sample may be divided into the weight changes resulting from *K*_1_ and *K*_2_ as:

14 $${\rm{\Delta }}{m}_{total}^{o}=x\cdot {m}_{K1}+(1-x)\cdot {m}_{K2}$$

where ${\rm{\Delta }}{m}_{total}^{o}$, *m*_*K*1_, and *m*_*K*2_ are the total weight change of the sample, weight change for the *K*_1_ reaction, and weight change for the *K*_2_ reaction, respectively. The weight change occurs by the *K*_2_ reaction at *x* = 0. Meanwhile, the weight change by the *K*_1_ reaction occurs at *x* = 1. For *x* < 1, the reaction of the consumption of holes is dominant by the *K*_2_ reaction; for *x* > 1, the formation of holes is dominant by the *K*_3_ (equation (7)) reaction. The overall nonstoichiometric water incorporation equation, combining *K*_1_ and *K*_2_, is denoted as:

15 $$\begin{array}{c}{K}_{4};{H}_{2}O+x{V}_{O}^{\cdot \cdot }+(2-x){O}_{O}^{\times }+(2-2x){h}^{\cdot }\\ \leftrightarrow 2O{H}_{O}^{\cdot }+(\frac{1-x}{2}){O}_{2}(g)\end{array}$$

The relationship between the variations in the concentration of each carrier and the measured weight change can be described as:

16 $${\rm{\Delta }}[j]=\frac{{\rm{\Delta }}{m}_{j}\cdot {M}_{s}}{{M}_{j}\cdot {m}_{s,0}}$$

where subscript *j* denotes species H_2_O, O (dry condition only) or H_2_; Δ*m*_*j*_ is the weight change of the specimen by the *j* species, *m*_*s*,0_ is the initial specimen weight, *M*_*j*_ is the molar weight of the *j* species and *M*_*s*_ is the molecular formula weight of the specimen.

The changes in the concentration of charge carriers are calculated using equation (16) during hydration (or dehydration). The four independent equations relating variations in the concentration of charge carriers should be satisfied when weight change occurs by water using TGA, as shown in the *K*_4_ reaction. The relationship between the structural elements under hydration at equilibrium is expressed below, using the changes in concentration of charge carriers causing weight change by the *K*_4_ reaction. The signal proceeds in the opposite direction under dehydration conditions.

17 $${\rm{\Delta }}[O{H}_{O}^{\cdot }]=2\cdot {\rm{\Delta }}[{H}_{2}O]$$

18 $${\rm{\Delta }}[{V}_{O}^{\cdot \cdot }]=-x\cdot {\rm{\Delta }}[{H}_{2}O]$$

19 $${\rm{\Delta }}[{O}_{O}^{\times }]=(x-2)\cdot {\rm{\Delta }}[{H}_{2}O]$$

20 $${\rm{\Delta }}p=(2x-2)\cdot {\rm{\Delta }}[{H}_{2}O]$$

## Results and Discussion

### Mass relaxation upon hydration/dehydration

**Figure 1:**
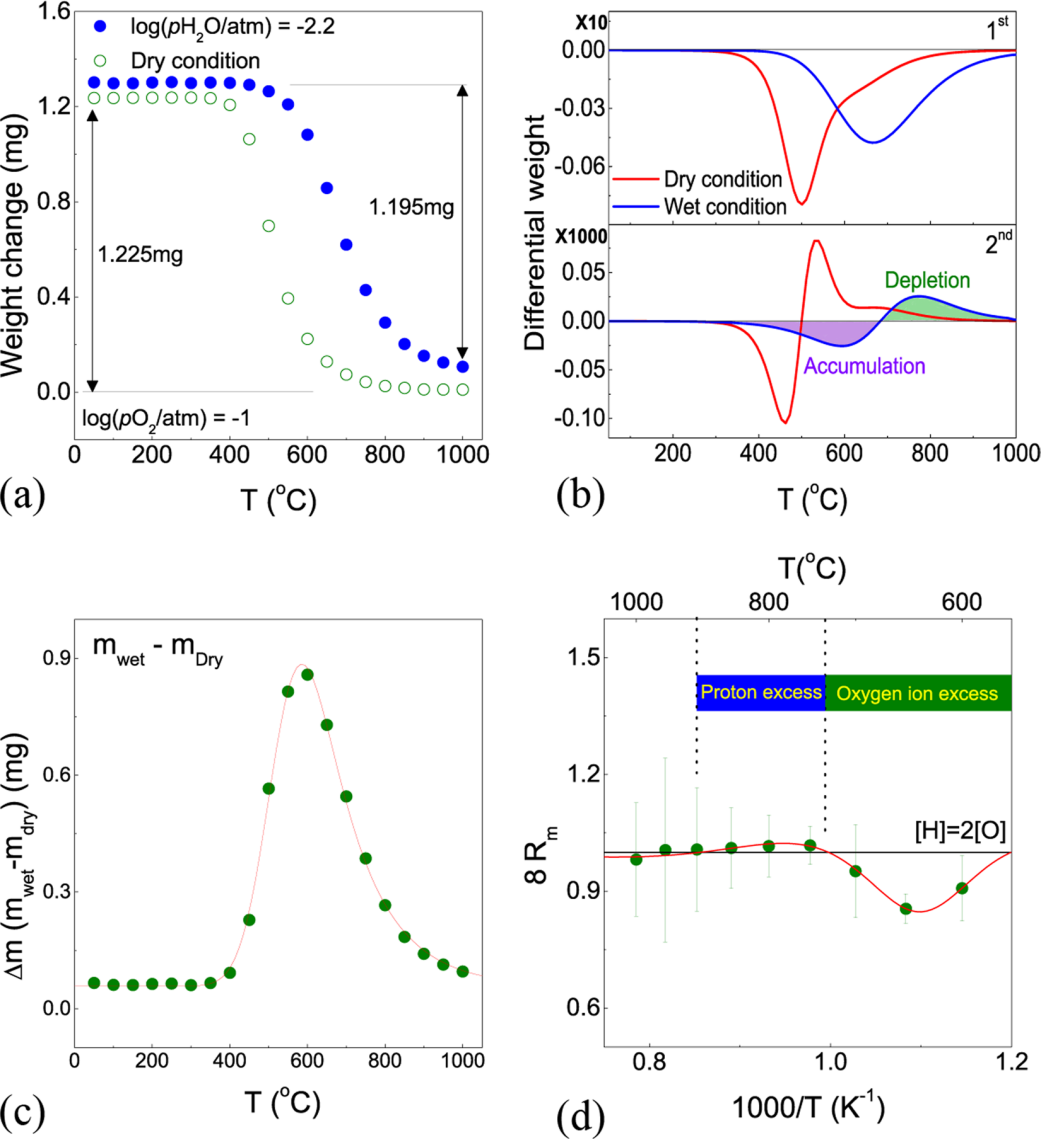
(**a**) Weight change, (**b**) differential weight of BCY10, (**c**) difference in weight between dry (*p*H_2_O = 4.0 × 10^−5^ atm) and wet (*p*H_2_O = 0.0063 atm) conditions, and (**d**) relative reaction concentrations, R_m_, using B_k_ (k = i, v) from TGA mass relaxation upon hydration as functions of temperature.

Figure 1a shows the weight change in dry (*p*H_2_O = 4.0 × 10^−5^ atm) and wet (*p*H_2_O = 0.0063 atm) conditions as functions of temperature after measuring the reference zero point of the weight under N_2_. The weight difference between the wet and dry conditions at 1000 °C indicates that it is caused by the reaction of *K*_4_. From the weight difference between the maximum and minimum temperatures, the total weight changes are 1.211 mg and 1.195 mg in wet and dry conditions, respectively. In terms of the low-temperature region in wet conditions, it looks that protons do not escape the oxide and instead remain present in the solid. The weight increases in both dry and wet conditions with decreasing temperature, while oxygen forms by filling the oxygen vacancies from the *K*_3_ reaction in dry conditions and the weight of oxide is increased by the *K*_4_ reaction in wet conditions. In the second-order derivative of the weight change as a function of temperature, as shown Fig. 1b, the maximum negative slope change occurs at ~460 °C for dry conditions and the maximum positive slope change appears at ~560 °C. The maximum point of the change in weight is at 500 °C as the maximized weight change by the *K*_3_ reaction. In wet conditions, the maximum negative and positive slope changes occur at ~600 °C and ~770 °C, respectively. The maximum point of the change in weight is ~670 °C, indicating that the weight change of the oxide appears most significantly under a given *p*H_2_O at this temperature. If the temperature increases above 670 °C, the magnitude of solubility of the water vapor reacting with the oxide is reduced.

The weight difference measured in dry and wet conditions is shown in Fig. 1c. The weight difference values indicate not only the largest differential of weights possibly combined into the oxide, but also the weight of water vapor incorporated for a given *p*H_2_O at each temperature. Because the weight change of the BCY10 sample occurs with significant dependence on the existing water vapor at ~600 °C, the thermal expansion coefficient (TEC) of the hydration state is assumed the largest. In addition, the value can be inferred to indicate the dependence of temperature on *p*H_2_O, because it is the difference in weight between the hydrated and dried specimens. Similar to these predictions, using *in*-*situ* high-temperature XRD equipment, Anderson *et al*.^19^ reported on the proton-conductor material BaCe_0.8_Y_0.2_O_2.9_ (BCY20) that the volume of a specimen rapidly changed near 600 °C at *p*H_2_O = 1 × 10^−4^ atm and that the temperature range in which the volume change occurred was increased with increasing *p*H_2_O.

To confirm the reaction of hydration with the sample as a direct proportion (H_2_O) from decoupled proton and oxygen, 8*R*_*m*_ as a function of temperature is shown in Fig. 1d using *B*_*k*_ (*k* = *i*, *v*) in equation (2), which is calculated from the mass relaxation. As expressed in equation (12), 8*R*_*m*_ is always equal to 1 if the stoichiometric hydration or dehydration reaction occurs by *K*_1_. 8*R*_*m*_ denotes the rate of concentration change in the reaction involving hydrogen (H_2_) and oxygen (O) when different partial pressures of water vapor are applied to the oxide. As seen in Fig. 1d, because 8*R*_*m*_ is below 1 in the region of 600–700 °C, oxygen is incorporated more into the oxide in the hydration reaction than hydrogen is. 8*R*_*m*_ is slightly greater than 1 above 750 °C in the high-temperature region, indicating that hydrogen becomes more involved in the hydration reaction to excess. The ratio of the concentration of hydrogen and oxygen reacting at ~1000 °C is almost 2:1, because the reaction may occur by the *K*_1_ mode with the extremely low hole concentration. Note that this value does not represent the absolute value of the solubility, indicating the ratio of the hydrogen and oxygen. From this result, the actual ion concentration participating in the hydration reaction has clearly different ratios with dependences on the thermodynamic conditions.

### Non-stoichiometry and concentration of charge carrier

The stoichiometric value (ABO_2.95_) is assumed to exist in the dry N_2_ atmosphere at 1000 °C; an oxygen non-stoichiometric value of 2.9503 is measured at *p*O_2_ = 0.1 atm at 1000 °C. Considering the main point defects of *p*-type BCY10 under wet conditions, the total charge-neutral conditions from Supplementary equation (S4) can be represented as:

21 $$2[{V}_{O}^{\cdot \cdot }]+[O{H}_{O}^{\cdot }]+p=[{Y}_{Ce}^{/}]$$

**Figure 2:**
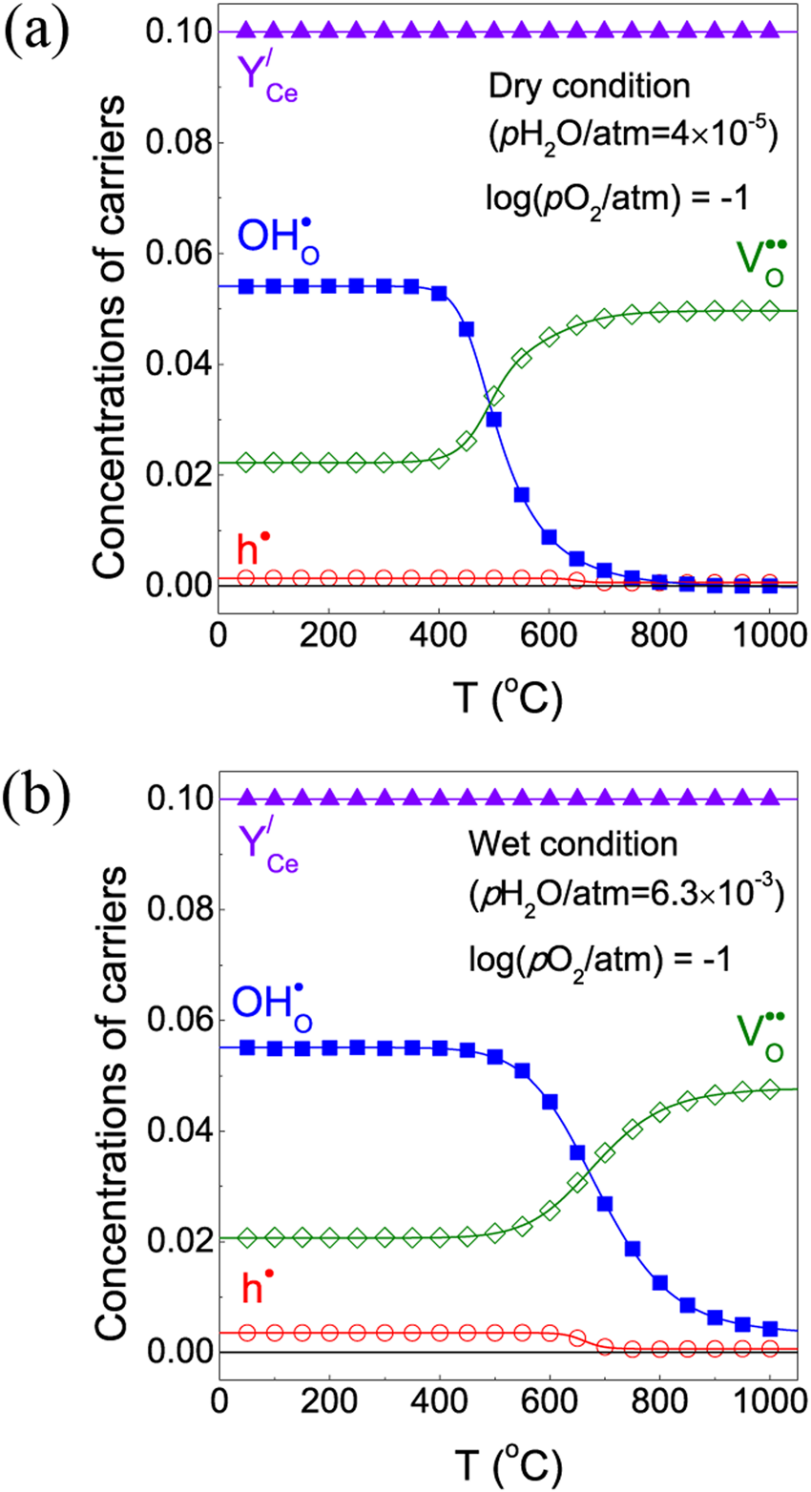
Concentrations of charge carriers under (**a**) dry condition (*p*H_2_O = 4.0 × 10^−5^ atm) and (**b**) wet condition (*p*H_2_O = 0.0063 atm).

Oishi *et al*.^20^ reported that the ionic valence of Ce^4+^ did not change with variations in temperature or *p*O_2_ in 10 mol% neodymium-doped BaCeO_3_ material at p-type conducting regime. Thus, the weight change with temperature at a fixed *p*O_2_ value is attributed only to redox reactions without involving cation reduction at lattice. Figure 2 shows the variations of concentration of each charge carrier species under dry and wet conditions. After the proton concentration sharply increases with a decrease in temperature, it tends to converge at lower temperatures. By contrast, the oxygen vacancy concentration is decreased by the reaction *K*_4_, but the concentration of holes tends to increase slightly at temperatures below 700 °C. The concentrations of the charge carriers satisfy the neutral condition of the hydration reaction expressed in equation (21) when the doping content is equal to 0.1. In Fig. 2b, the concentrations of charge carriers change rapidly within the temperature range of 600–800 °C. For typically known BCY-BZY proton conductor materials, non-linear thermal expansion properties have been reported within specific temperature ranges for each condition^19, 21, 22^. In particular, Lvagaeva *et al*.^23^ reported that the TEC of the BCY proton conductor between 600–800 °C showed a drastic change, similar to the alteration in the concentrations of charge carriers in this work. Consequently, the sudden variations of concentrations in particular temperature regions may have affected the expansion of the specimen. From this result regarding the concentration of charge carriers, the concentrations of holes and protons have low values at high temperatures, coinciding with the reason for the vanishing two-fold relaxation profile with increasing temperature, as mentioned above.

**Figure 3:**
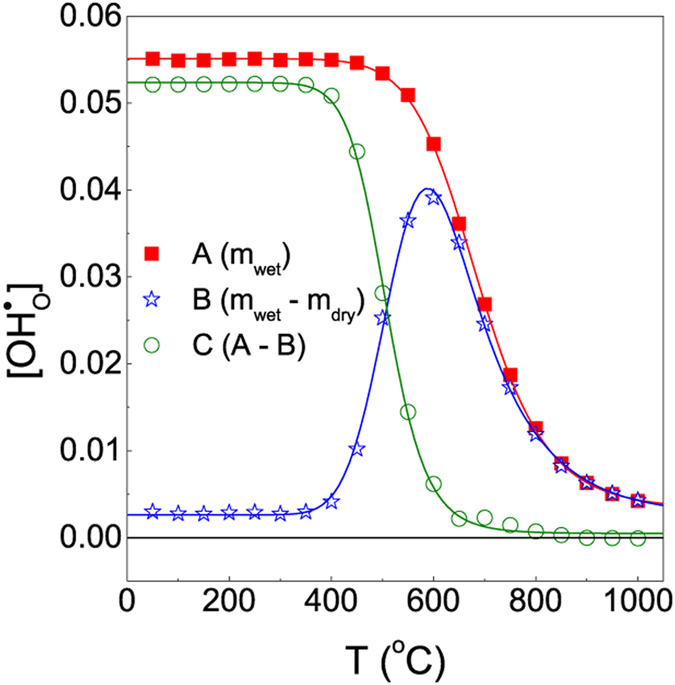
Generated proton concentration under wet condition (A-line), proton concentration caused by condition between dry and wet (B-line), and difference of proton concentration between A- and B- line (C-line).

Figure 3 shows the generated proton concentration with decreasing temperature (A-line), the proton concentration caused by the weight difference between the dry (*p*H_2_O = 4.0 × 10^−5^ atm) and wet (*p*H_2_O = 0.0063 atm) conditions (B-line), and the difference of proton concentration between the A- and B-lines (C-line). Because the C-line shows the difference of proton concentration between the existing protons in the oxide and the protons that can enter the sample when the dry condition is changed to a wet condition, the C-line value indicates the proton concentration remaining in the specimen. Examining the variation in proton concentration with decreasing temperatures in the wet condition in the curve of the A-line, the proton concentration in the sample increases steadily as the temperature decreases to reach the value of 0.055; this is maintained at low temperatures. In the B-line curve, the part with constant proton concentration in the initial low-temperature region (below 400 °C) is caused by the slow reaction rate from the low temperature; the proton concentration is increased by thermal excitation in the middle temperature (400–600 °C) region. The proton concentration tends to decrease with the decreasing solubility of protons, which occurs in the specimen over 600 °C. This phenomenon is similar to that reported previously^24^, in which it was interpreted as the free-moving protons (trap-free) and those trapped in the specimen for the Y-doped BaZrO_3−δ_ proton conductor. The thermodynamically predicted proton concentration is equal to the amount of protons generated by the dry-wet change in each temperature range, because the hydration reaction proceeds at a sufficiently high rate in the high-temperature zone above 800 °C as shown in the A- and B-line curves in Fig. 3. However, the amount of protons remaining in the specimen increases at lower temperatures because of the increasing difference between the A- and B-line proton concentrations. Because the protons in trapped state cannot escape the specimen with decreases in temperature, the protons may accumulate in the oxide.

### Equilibrium constant of hydration reactions

**Figure 4:**
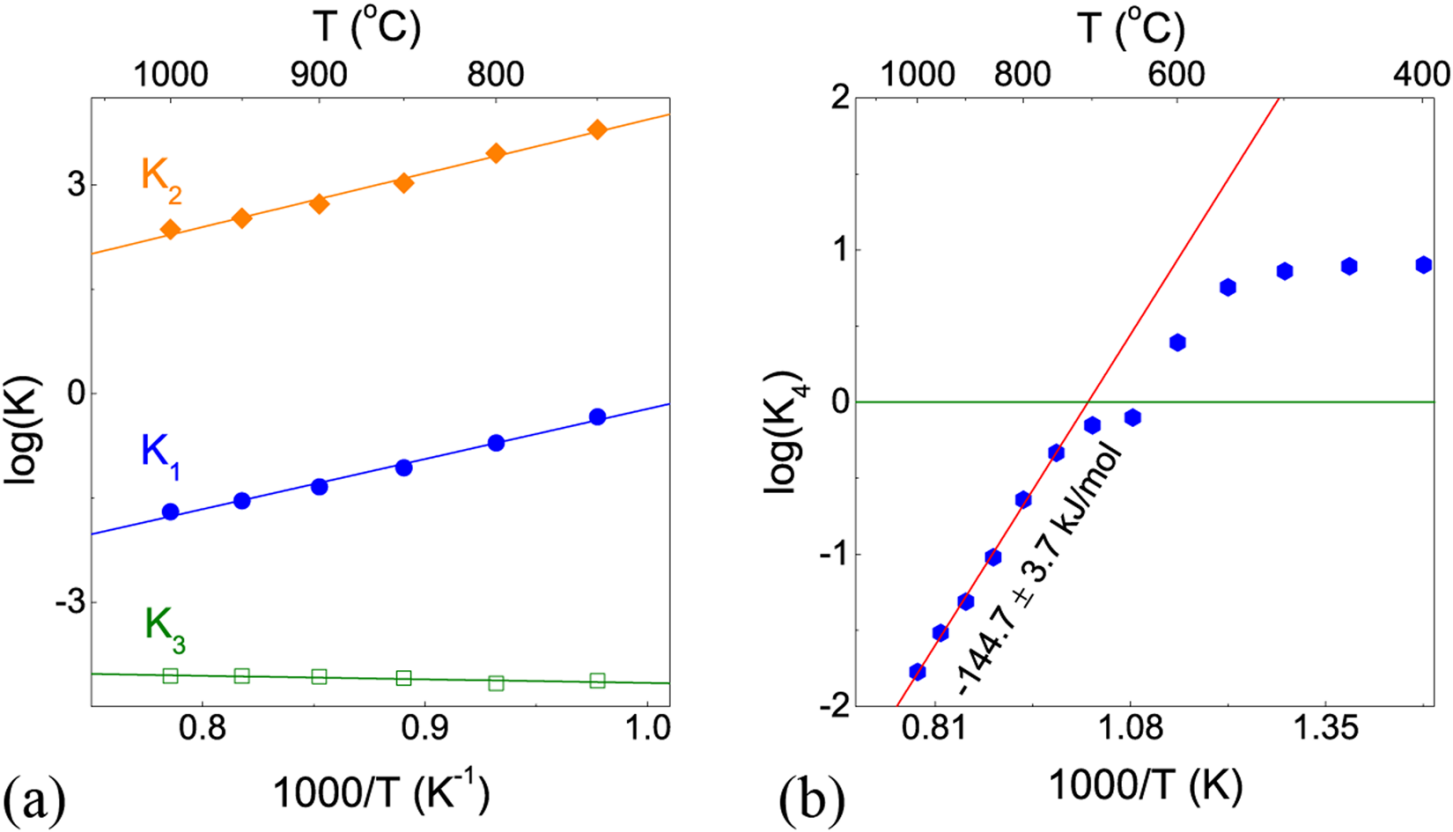
Equilibrium constants (**a**) of K_1_, K_2_, and K_3_ reactions and (**b**) of K_4_ reactions.

Figure 4a shows each reaction equilibrium constants for the high-temperature region (750–1000 °C). For *K*_1_ reaction, dehydration occurs above the 700 °C boundary. For *K*_2_, the reaction to produce protons is predominant in the range of measured temperatures, but the hole concentration generated by the *K*_2_ reaction is small because of the relatively trivial proton concentration in the high-temperature region. The reduction reaction is dominant in the *K*_3_ reaction. Reviewing the overall reaction (*K*_4_) by combining *K*_1_ and *K*_2_, as seen in Fig. 4b, the dehydration reaction dominates at temperatures above ~600 °C; in contrast, hydration dominates at temperatures below ~600 °C under *p*H_2_O = 0.0063 atm and *p*O_2_ = 0.1 atm. These results showed the same results mentioned above: the maximum solubility by hydration is shown at 600 °C as the maximum weight change, and the solubility of water vapor is decreased with increasing temperature from the boundary of ~600 °C. The enthalpy and entropy are compared to literature reports^6, 7, 25–29^ in Table 1 and these values for *K*_1_ appear to be reasonable when compared with the entropies and enthalpies of similar materials. The newly extracted enthalpy and entropy of the *K*_4_ reaction are shown as −144.7 ± 3.7 kJ/mol and −147.8 ± 3.2 J/mol · K, respectively.

**Table 1:** The equilibrium constants for each reaction in high-temperature region (750–1000 °C).

Materials	Type	*H* (kJ/mol)	*S* (J/mol · K)	References
BaCe_0.9_Y_0.1_O_3−δ_	*K*_*1*_	−138.0 ± 6.9	−142.2 ± 6.1	This work*
BaCe_0.9_Y_0.1_O_3−δ_	*K*_*2*_	−147.8 ± 8.2	−72.4 ± 7.2	This work*
BaCe_0.9_Y_0.1_O_3−δ_	*K*_*3*_	9.8 ± 3.0	−69.8 ± 2.6	This work*
BaCe_0.9_Y_0.1_O_3−δ_	*K*_*4*_	−144.7 ± 3.7	−147.8 ± 3.2	This work*
BaCe_0.9_Y_0.1_O_3−δ_	*K*_*1*_	−122	−119	28
BaCe_0.9_Y_0.1_O_3−δ_	*K*_*1*_	−163.3	−167.9	6
BaCe_0.9_Y_0.1_O_3−δ_	*K*_*1*_	−123	−113	29
BaCe_0.9_Yb_0.1_O_3−δ_	*K*_*1*_	−127	−126	28
BaCe_0.6_Zr_0.3_Y_0.1_O_3−δ_	*K*_*1*_	−106	−104	29
BaCe_0.2_Zr_0.7_Y_0.1_O_3−δ_	*K*_*1*_	−93	−96	29
BaZr_0.9_Y_0.1_O_3−δ_	*K*_*1*_	−79.5	−88.9	6
BaZr_0.9_Y_0.1_O_3−δ_	*K*_*1*_	−83.3	−91.2	29
SrCe_0.95_Eu_0.05_O_3−δ_	*K*_*1*_	−164	—	26
SrCe_0.95_Yb_0.05_O_3−δ_	*K*_*1*_	−157	−128	7
SrTi_0.98_Sc_0.02_O_3−δ_	*K*_*1*_	−21.7	−97.5	6
Ba_3_Ca_1.17_Nb_1.83_O_9−δ_	*K*_*1*_	−65.2	−103.7	6
Ba_3_Ca_1.18_Nb_1.82_O_9−δ_	*K*_*1*_	−78.5	−111	27
Ba_0.5_Sr_0.5_Fe_0.8_Zn_0.2_O_3−δ_	*K*_*1*_	−70	−150	25
Ba_2_YSnO_5.5_	*K*_*1*_	−79.9	−108.8	6

*The value of “This work” is calculated with temperature range of 1000–750 °C.

As shown in Fig. 2, the proton concentration decreases with temperature above 500 °C, suggesting exothermic reaction with negative activation energy of *K*_1_, *K*_2_, and *K*_4_ at given in Fig. 4, which is the very general trend of proton conducting perovskites. On the contrary, positive oxidation enthalpy in Fig. 4 was expected from the increase of oxygen vacancy concentration at temperature above 750 °C in Fig. 2, leading to endothermic oxidation reaction. This results are opposite to the DFT calculation that the exothermic oxidation reaction may be unfavorable at high temperature while favored at low temperature^30^. However, one should note another recent report saying that the sign of oxidation enthalpy may be influenced by the deep acceptor levels of wide-band-gap acceptor-doped perovskites^31^. The further discussion regarding the oxidation enthalpy contribution involving from cerium oxidation variation and/or acceptor levels within band gap should be discussed with more systematic data in future.

## Conclusions

A mass relaxation experiment on Y-doped BaCeO_3_ was performed below 1000 °C in the *p*-type regime to understand clearly the phenomenon of decoupled hydrogen and oxygen, which causes the two-fold conductivity relaxation profile. The decoupled ions participated in hydration/dehydration reactions were proven to be at different ratios from the result introduced by the 8*R*_*m*_ function, depending on the thermodynamic conditions. Oxygen species were more involved than hydrogen from water in the oxide between 600–700 °C, while hydrogen was slightly active to excess above 750–900 °C in the hydration reaction. In the weight difference between the dry and wet conditions, the total weight change in the wet condition was lower than that in the dry condition, indicating that protons absorbed in the wet condition remained in the oxide as the temperature decreased. Therefore, the proton concentration tended to converge at low temperatures to reach a value of 0.055 at *p*H_2_O = 0.0063 atm and *p*O_2_ = 0.1 atm. Each reaction equilibrium constant was calculated in the high-temperature region of 750–1000 °C; the enthalpy and entropy of the nonstoichiometric hydration reaction are −144.7 ± 3.7 kJ/mol and −147.8 ± 3.2 J/mol · K, respectively.

## Method

### Sample preparation

Polycrystalline BaCe_0.9_Y_0.1_O_3−δ_ (BCY10) powders were prepared by a solid-state reaction method, with BaCO_3_ (purity 99.99%), CeO_2_ (purity 99.99%), and Y_2_O_3_ (purity 99.99%) used as starting materials, purchased from Alfa Aesar and Sigma Aldrich. The starting materials were mixed in stoichiometric amounts and ball-milled with stabilized zirconia balls and isopropyl alcohol for 24 h. The mixed powder was dried in an oven at 80 °C for 10 h and the mixture was subsequently calcined at 1300 °C in air for 10 h to get BCY10. The calcined BCY10 was planetary ball-milled with stabilized zirconia balls and isopropyl alcohol at 300 rpm for 4 h in order to obtain a fine powder by crushing large-sized particles. The calcined powder was molded into a bar, cold-pressed isostatically at 150 MPa, and sintered at 1600 °C for 10 h in air atmosphere after covering with same powder. The sintered sample was cut by a low-speed saw into a rectangular parallelepiped specimen. A polisher was used to control the evenness to a level below 1 µm using various sandpapers and chemical abrasives. The sizes of the samples used for the mass relaxation and two-fold relaxation experiments were 0.42 × 0.54 × 0.58 cm^3^ (weight = 0.7944 g) and 0.24 × 0.24 × 1.49 cm^3^, respectively.

### Thermogravimetric analysis measurement

Before the TGA measurements, the specimen was baked under flowing dry gas for 24 h at 1000 °C to fully remove the guest protons which can be present in oxides. The dry gas was passed through a coiled cold trap zone of about 1 m in length to thoroughly remove any water vapor present in the gas. The change in weight was monitored during the baking process; the weight maintained a constant value for 10 h within the resolution range (±1 µg) of the TGA apparatus. After baking, measurement was performed for decreasing temperatures in steps of 50 °C from 1000 °C. Wet-condition baking was performed by flowing wet gas (*p*H_2_O = 6.3 × 10^−3^ atm) for 10 h while monitoring the weight of the specimen till the weight becomes constant within the resolution range of the TGA equipment. After this determination, the experiment was performed under the same temperature step conditions as in the dry case (*p*H_2_O = 4.0 × 10^−5^ atm). During this experiment, when all given conditions were fixed excepting temperature, the analysis of weight change as a function of time can be difficult to interpret in low-temperature range because the reaction rate related to the weight change quantity, is lower at the lower temperatures. Therefore, to minimize the error from the lower reaction rate, a stabilization process are performed by holding the sample, at lower temperatures (below 700 °C), for ~10–100 h and, at high temperatures (exceeding 700 °C), for ~2–10 h.

## Electronic supplementary material

Supplementary information
