# Supplementary material for: Hydration of Proton-conducting BaCe0.9Y0.1O3−δ by Decoupled Mass Transport
Source: Sci Rep. 2017 Mar 28;7:486. doi: 10.1038/s41598-017-00595-w (PMC5428669; doi:10.1038/s41598-017-00595-w)
Supplement: Supplementary file 1 — Supplementary information [file 41598_2017_595_MOESM1_ESM.pdf]

Supplementary Information

Hydration of Proton-conducting $BaCe_{0.9}Y_{0.1}O_{3-\delta}$ by Decoupled Mass Transport

Dae-Kwang Lim [a], Ha-Ni Im [a], Sun-Ju Song[a,†] and Han-Ill Yoo[b]

[a]Department of Materials Science and Engineering, Chonnam National University, Gwangju, 61186, Republic of Korea.

[b]Department of Materials Science and Engineering, Seoul National University, Seoul, 08826, Republic of Korea.

[†]Corresponding author:

Professor Sun-Ju Song

Department of Materials Science and Engineering

Chonnam National University, South Korea

Email: song@chonnam.ac.kr

Theoretical background

In Y-doped $BaCeO_3$ oxide, the substitution of Ce^{4+} by Y^{3+} leads to the formation of oxygen vacancies, as represented below using Kröger–Vink notation[1]:

$$\boldsymbol{Y_2O_3 \xrightarrow{BaCeO_3} Y_{Ce}^{/} + O_O^X + \frac{1}{2}V_O^{\cdot\cdot}} \quad \text{(S1)}$$

The oxygen vacancies ($\boldsymbol{V_O^{\cdot\cdot}}$) facilitate oxygen–ion conduction and promote the formation of holes in oxidizing conditions, as represented below:

$$\boldsymbol{\frac{1}{2}O_2(g) + V_O^{\cdot\cdot} \leftrightarrow O_O^X + 2h^{\cdot}; \quad K_{OX} = \frac{[O_O^{\times}]p^2}{[V_O^{\cdot\cdot}](pO_2)^{1/2}}} \quad \text{(S2)}$$

Similarly, in a hydrogen-containing atmosphere, e.g., in a humid atmosphere, the oxygen vacancies can act as proton incorporation sites, as represented below:

$$\boldsymbol{H_2O(g) + V_O^{\cdot\cdot} + O_O^{\times} \leftrightarrow 2OH_O^{\cdot}; \quad K_W = \frac{[OH_O^{\cdot}]^2}{[V_O^{\cdot\cdot}][O_O^{\times}](pH_2O)}} \quad \text{(S3)}$$

where $\boldsymbol{K_{Ox}}$ is the equilibrium constant for oxygen exchange, *p* is the concentration of holes, $\boldsymbol{K_w}$ is the equilibrium constant for the exchange of water, and the species $\boldsymbol{OH_O^{\cdot}}$ represents a proton attached to a lattice oxygen atom by hydrogen bonding.

The charge-neutrality condition can be expressed by the following equation:

$$\mathbf{n} + \boldsymbol{[Y_{Ce}^{/}] = p + 2[V_O^{\cdot\cdot}] + [OH_O^{\cdot}]} \quad \text{(S4)}$$

The proton and oxygen vacancies may diffuse into the oxides by downhill diffusion against partial-pressure gradients because of the relative rate of diffusion in *p*-type oxidizing regime. The fluxes at diffusion controlled conditions during hydration or dehydration in a fixed oxygen-activity atmosphere can be given as:

$$J_i = -\frac{\sigma_{H^+}(1-t_{H^+})}{2F^2}\nabla\mu_{H_2O} = -\widetilde{D}_{iH}\nabla C_i \quad \text{(S5)}$$

$$J_v = -\frac{\sigma_{O^{2-}}t_{H^+}}{4F^2}\nabla\mu_{H_2O} = -\widetilde{D}_{vH}\nabla C_v \quad \text{(S6)}$$

where J_i denotes the permeation of hydrogen, J_v the permeation of oxygen, and σ_k and t_k the partial electrical conductivity and transference number of *k*-type carriers (*k* = O^{2-}, H^+, and h^+), respectively. The two chemical diffusivities, or the hydrogen and oxygen diffusivities, may be defined as[2]:

$$\widetilde{D}_{iH} = \frac{\sigma_{H^+}(1-t_{H^+})}{2F^2}\left|\frac{\partial\mu_{H_2O}}{\partial C_i}\right|_{\nabla\mu_{O_2}=0} \quad \text{(S7)}$$

$$\widetilde{D}_{vH} = \frac{\sigma_{O^{2-}}t_{H^+}}{4F^2}\left|\frac{\partial\mu_{H_2O}}{\partial C_v}\right|_{\nabla\mu_{O_2}=0} \quad \text{(S8)}$$

where the quantities within the absolute-value signs (| |) are the corresponding thermodynamics factors, C_i is the proton concentration, and C_v the oxygen vacancy concentration. Here, $\widetilde{D}_{iH}$ and $\widetilde{D}_{vH}$ may be interpreted as the chemical diffusion coefficients of hydrogen and oxygen as a function of pH$_2$O at a fixed oxygen partial pressure.

By changing the water vapor pressure, the thermodynamic equilibrium shifts to a new condition with time and accordingly the changes in concentration of $OH_O^{\cdot}$ and $V_O^{\cdot\cdot}$ over time may be written as:

$$\frac{\partial C_k}{\partial t} = \widetilde{D}_{kH}\nabla^2 C_k\Big|_{\nabla\mu_O=0} \quad (k = i, v) \quad \text{(S9)}$$

The chemical diffusivity ($\widetilde{D}_{kH}$) is assumed to remain constant throughout the interior of the specimen, notwithstanding a difference in water vapor pressure between the inside and outside

of the specimen. A general solution of Supplementary equation (S9), relating to the difference in the total average concentration of each charge carrier, is expressed as:

$$\overline{C}_k - C_{k,0} = (C_{k,\infty} - C_{k,0})[1 - f(\tau_k)] \tag{S10}$$

where $C_{k,0}$ and $C_{k,\infty}$ are the initial and final concentrations of each charge carrier, $k = (i, v)$, at $t = 0$ and $t = \infty$, respectively.

Solving Fick's second law for each defect concentration with appropriate initial and boundary conditions, produces an analytic solution for conductivity relaxation in closed form for a two-dimensional $2a \times 2a$ geometry as follows[3,4]:

$$\sigma(t) = P_0 + P_i f_i(\widetilde{D}_{iH}, t) - P_v f_v(\widetilde{D}_{vH}, t) \tag{S11}$$

with

$$f_k(\widetilde{D}_{kH}, t) = \left[\sum_{n=1}^{\infty} \frac{2(\beta_n tan\beta_n)^2 exp\left(-\frac{\beta_n^2 \widetilde{D}_{kH} t}{a^2}\right)}{\beta_n^2(\beta_n^2 + (\beta_n tan\beta_n)^2 + \beta_n tan\beta_n)}\right]^2 \tag{S12}$$

where β_n is the positive root of $\beta \tan \beta = L$; $L = ak/\widetilde{D}$ and P_k $(k = 0, i, v)$ are constants related to the mobilities and initial and final concentrations of the defects involved, respectively.

Experimental

Physical characterization

The obtained powders were characterized by X-ray diffraction (XRD; D/MAX Ultima III, Rigaku, Japan) equipped with a Cu target X-ray tube at a scan rate of 2°/min between scanning angles (2θ) of 10-80°. The patterns were refined using a profile-matching method. The microstructure of the sintered body was analyzed by scanning electron microscopy (SEM; Shimadzu, SS-550).

Relaxation measurements

The ECR and mass relaxation were recorded as function of time with respect to the variations in various thermodynamic parameters, using a standard four-probe DC-conductivity measurement method and thermogravimetric analysis (TGA), respectively. The ECR and mass relaxation measurements were performed under conditions of varying humidity ($-3.0 \leq \log(pH_2O/\text{atm}) \leq -1.6$) by a Dewpoint meter (DMP74A) at a fixed oxygen partial pressure ($\log(pO_2/\text{atm}) = -1.00 \pm 0.01$).

Results and discussion

Physical characterization

Supplementary Figure S1a shows the XRD pattern and refined profile-matched data of the crushed BCY10 powder after sintering at 1600 °C for 10 h. The XRD patterns show that all the peaks can be assigned to an orthorhombic lattice of Y-doped barium cerate[5], indicating that the sintered BCY10 powders have high purity. The orthorhombic (P*mcn*) lattice parameters are a = 8.831, b = 6.325, and c = 6.242 Å. Supplementary Figure S1b shows SEM image of the fractured cross-section of a BCY10 sintered pellet. The sample is sufficiently sintered with negligible porosity and a relative density, as measured by the Archimedes method, is >96 %.

Conductivity relaxation upon hydration/dehydration

Supplementary Figure S2 depicts typical relaxation profiles from DC four-probe conductivity relaxation and experimental TGA weight change data under identical measurement conditions ($\log(pO_2/\text{atm}) = 0.1$; $\log(pH_2O/\text{atm}) = -2.4 \leftrightarrow -2.2$). The two-fold conductivity relaxation profile and monotonic TG mass relaxation profile have different aspects of the imposed water potential gradient at a constant oxygen partial pressure. As shown in Supplementary Figure S2a, non-monotonic conductivity relaxation is interpreted by the incorporation or dissociation of hydrogen and oxygen components with the oxide or by the ambipolar diffusion of protons ($\boldsymbol{OH_O^{\cdot}}$ or $\boldsymbol{H_i^{\cdot}}$) with holes ($\boldsymbol{h^{\cdot}}$) and of oxygen vacancies ($\boldsymbol{V_O^{\cdot\cdot}}$) with holes, illustrating a competition between two chemical diffusion processes. However, the weight change experiment shows a monotonic relaxation profile, as seen in Supplementary Figure S2b, because the concentration of hydrogen and oxygen incorporated during the hydration increase with increasing the weight of the oxide; the opposite trend is observed in dehydration.

The two-fold conductivity relaxation profile becomes monotonic as the temperature is increased, as shown in Supplementary Figure S3; this can be attributed to three plausible reasons. First, the concentration of holes is reduced with increasing the degree of reduction of specimen. Kim and Yoo[6] reported that the two-fold profile changed to a monotonic profile as the oxygen partial pressure decreased in a constant thermodynamic condition. The monotonic relaxation can arise from identical values of the chemical diffusivity of protons ($\tilde{\boldsymbol{D}}_{\boldsymbol{iH}}$) and oxygen vacancies ($\tilde{\boldsymbol{D}}_{\boldsymbol{vH}}$), with a minor hole-transference number in reducing conditions[6,7]. Second, when chemical diffusion of oxygen ions occurs much faster at high temperatures and, in other conditions, a monotonic relaxation may occur even if diffusion proceeds with decoupled hydrogen and oxygen species. A previous study[8] reported that monotonic relaxation could appear when oxygen diffusion is sufficiently faster compared with proton diffusion, while reverse two-fold conductivity relaxation could appear when $\tilde{\boldsymbol{D}}_{\boldsymbol{vH}}$ is three times higher than $\tilde{\boldsymbol{D}}_{\boldsymbol{iH}}$. Third, when the proton conductivity contribution to the total conductivity is negligible by decreasing the proton concentration in specimen as the temperature increases to a high level, which leads to thermodynamically unstable protons. The current phenomenon of the monotonic conductivity relaxation profile at high temperatures can be explained by a combination of the above reasons.

Supplementary Figure S4 shows the proton and oxygen chemical diffusivity as functions of temperature and water vapor pressure, calculated by nonlinear square fitting of Supplementary equation (S11) and equation (2) at constant oxygen partial pressure ($\log(pO_2/\text{atm}) = -1.00 \pm 0.01$) to mass relaxation and two-fold conductivity relaxation profiles. The oxygen partial pressure was maintained at 0.100 atm within an acceptable error range (±0.002 atm) while the water vapor pressure was changed within 1.5 orders. Consequently, the oxygen chemical diffusivity may appear to be independent of the water vapor pressure. However, as seen in Supplementary Figure S4, although oxygen chemical diffusivities are scattered, they decrease

slightly with increases in pH_2O. This may be related to the reduction of oxygen vacancies by the increasing incorporation of H_2O from the K_4 reaction (equation (15)). In addition to this change in oxygen diffusivity, the proton concentration and proton chemical diffusivity are increased with increasing pH_2O, which reacts with the sample by the K_4 reaction. From the two chemical diffusivities, the increase in proton concentration is higher than the decrease in the oxygen vacancy concentration at a constant temperature with a rising pH_2O. The proton and oxygen chemical diffusivity, determined under the same thermodynamic conditions and calculated from two experiments with different physical properties, are given nearly equal values within an error range. The measured proton and oxygen chemical diffusivity in ~700–850 °C temperature range of clearly observed two-fold relaxation are ~0.91×10^{-4}–6.53×10^{-4} and ~1.14×10^{-5}–4.84×10^{-5} $cm^2 \cdot s^{-1}$, respectively.

Supplementary Figure S5a shows the chemical diffusivity with changing pH_2O (0.016 atm ↔ 0.010 atm) at pO_2 = 0.1 atm via DC four-probe conductivity relaxation for ~700–900 °C; the points of 950 °C and 1000 °C show the monotonic relaxation. The activation energies for the proton and oxygen chemical diffusivity coefficients are 0.46 ± 0.04 eV and 0.82 ± 0.09 eV, respectively. Under the same conditions, as can be seen in Supplementary Figure S5b, the proton chemical diffusivity coefficient calculated from mass relaxation is higher than the oxygen chemical diffusivity coefficient for the range above 900 °C, although it has a relatively large error value. However, the activation energies of proton and oxygen chemical diffusivity coefficients are 0.38 ± 0.11 eV and 0.69 ± 0.07 eV, respectively, by the mass relaxation experiment. Consequentially, the activation energies of chemical diffusivity obtained from the two measurement systems agree within the error range. One reason for the monotonic relaxation profile in Supplementary Figure S3 may be the fast oxygen diffusion; the oxygen diffusion gradually increases with increasing temperature as the differences diminish between the proton and oxygen chemical diffusivity coefficients.

References

1. Kröger, F. A. & Vink, H. J. Relations Between the Concentrations of Imperfections in Crystalline Solids in *Solid State Physics* (ed. Camley R. E. & Stamps R. L.) 307-435 (Academic Press, 1956).
2. Yoo, H.-I. Kim, J.-K. & Lee, C.-E. Electrical Conductivity Relaxations and Chemical Diffusivities of $BaCe_{0.95}Yb_{0.05}O_{2.975}$ upon Hydration and Oxidation. *J. Electrochem. Soc.* **156**, B66-B73 (2009).
3. Yoo, H.-I. Yoon, J.-Y. Ha, J.-S. & Lee, C.-E. Hydration and oxidation kinetics of a proton conductor oxide, $SrCe_{0.95}Yb_{0.05}O_{2.975}$. *Phys. Chem. Chem. Phys.* **10**, 974-982 (2008).
4. Yoo, H.-I. & Lee, C.-E. Conductivity relaxation patterns of mixed conductor oxides under a chemical potential gradient. *Solid State Ion.* **180**, 326-337 (2009).
5. Takeuchi, K. et al. The Crystal Structures and Phase Transitions in Y-doped $BaCeO_3$: Their Dependence on Y Concentration and Hydrogen Doping, *Solid State Ion.* **138**, 63-77 (2000).
6. Kim, E. Yoo, H.-I. Two-Fold -to-Single-Fold Transition of the Conductivity Relaxation Patterns of Proton-Conducting Oxides upon Hydration/Dehydration, *Solid State Ion.* **252**, 132-139 (2013).
7. Poetzsch, D. Merkle, R. Maier, J. Proton Conductivity in Mixed-Conducting BSFZ Perovskite from Thermogravimetric Relaxation, *Phys. Chem. Chem. Phys.* **16**, 16446-16453 (2014).
8. Lim, D.-K. Singh, B. Choi, M.-B. Song, S.-J. Study of Hydration/Dehydration Kinetics of SOFC Cathode Material $Ba_{0.5}Sr_{0.5}Co_{0.8}Fe_{0.2}O_{3-\delta}$ by Electrical Conductivity Relaxation Technique, *J. Electrochem. Soc.* **160**, F764-F768 (2013).

Figure list

(a)

(b)

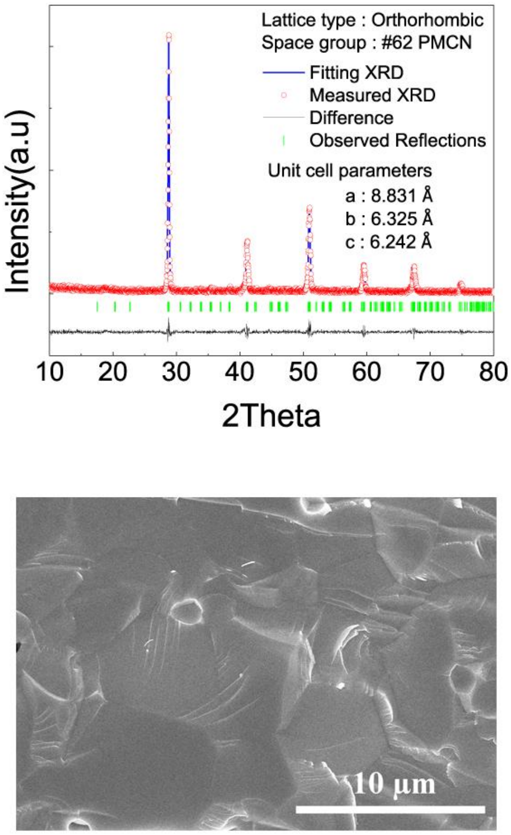

Supplementary Figure S1. (a) XRD pattern of BCY10 crushed powder and (b) SEM image of cross-section of BCY10 specimen sintered at 1600 °C for 10 h.

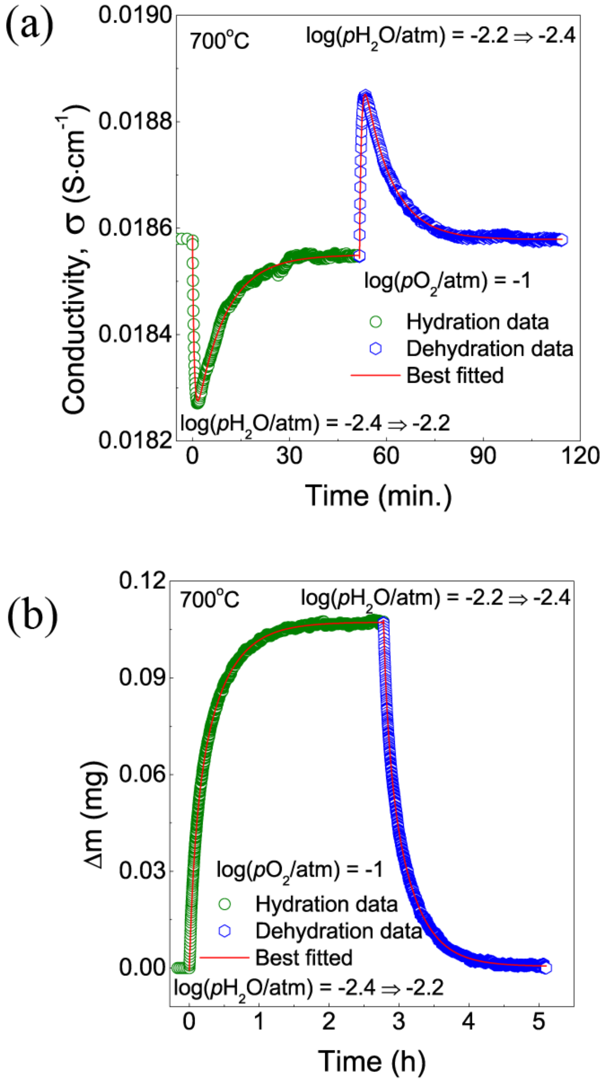

Supplementary Figure S2. (a) Typical two-fold conductivity and (b) mass relaxation profiles at log(pO_2/atm) = 0.1 and log(pH_2O/atm) = -2.2 ↔ -2.4.

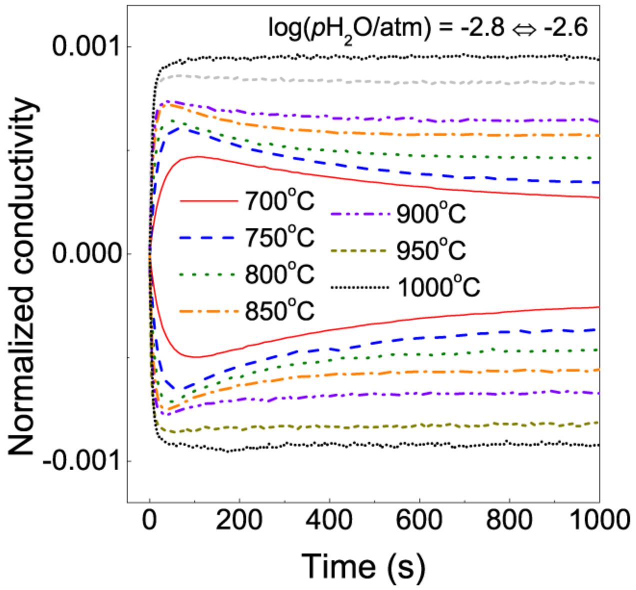

Supplementary Figure S3. Two-fold conductivity relaxation profiles as a function of temperature at log(pH_2O/atm) = -2.6 ↔ -2.8.

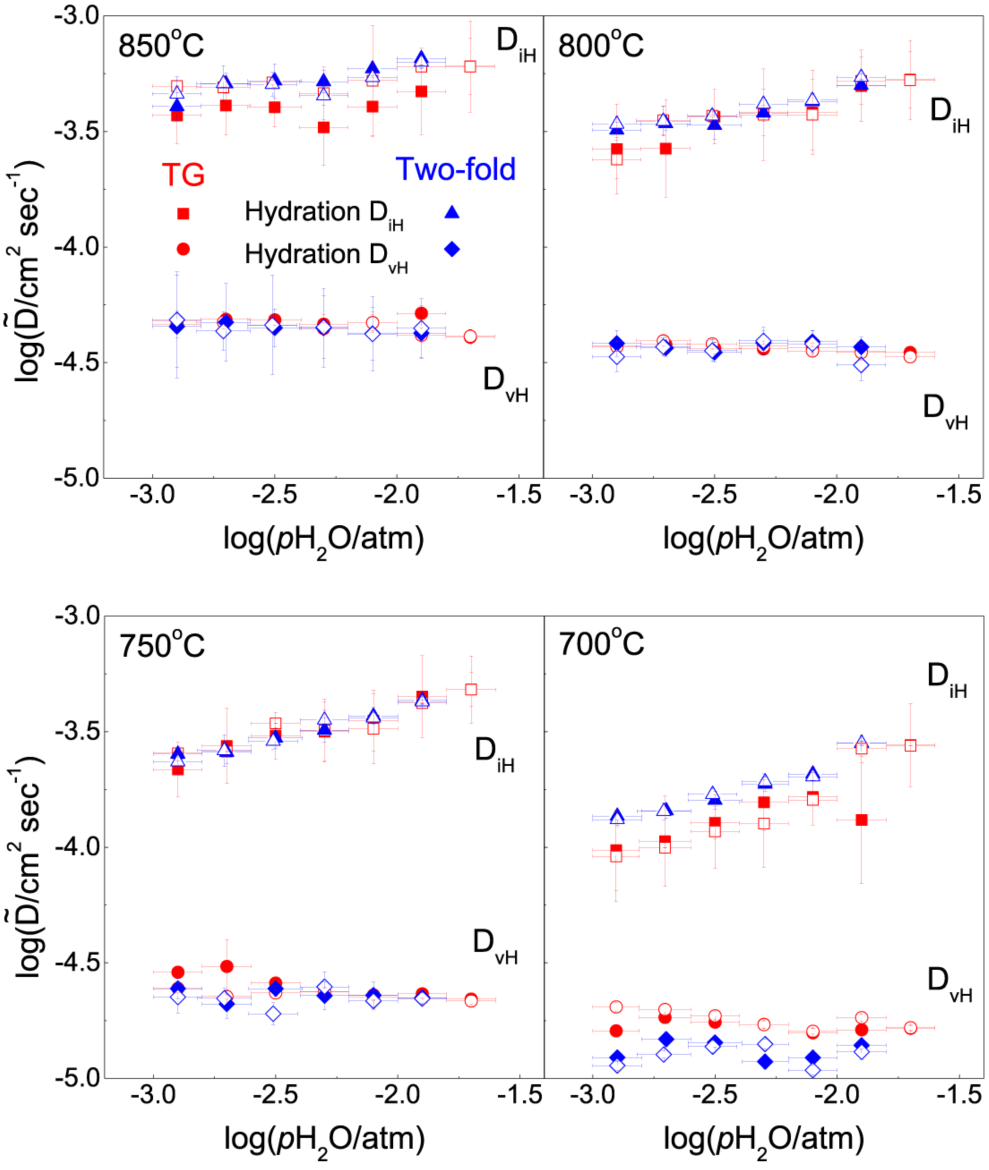

Supplementary Figure S4. Comparison of chemical diffusivity coefficients from TG mass relaxation and two-fold conductivity relaxation.

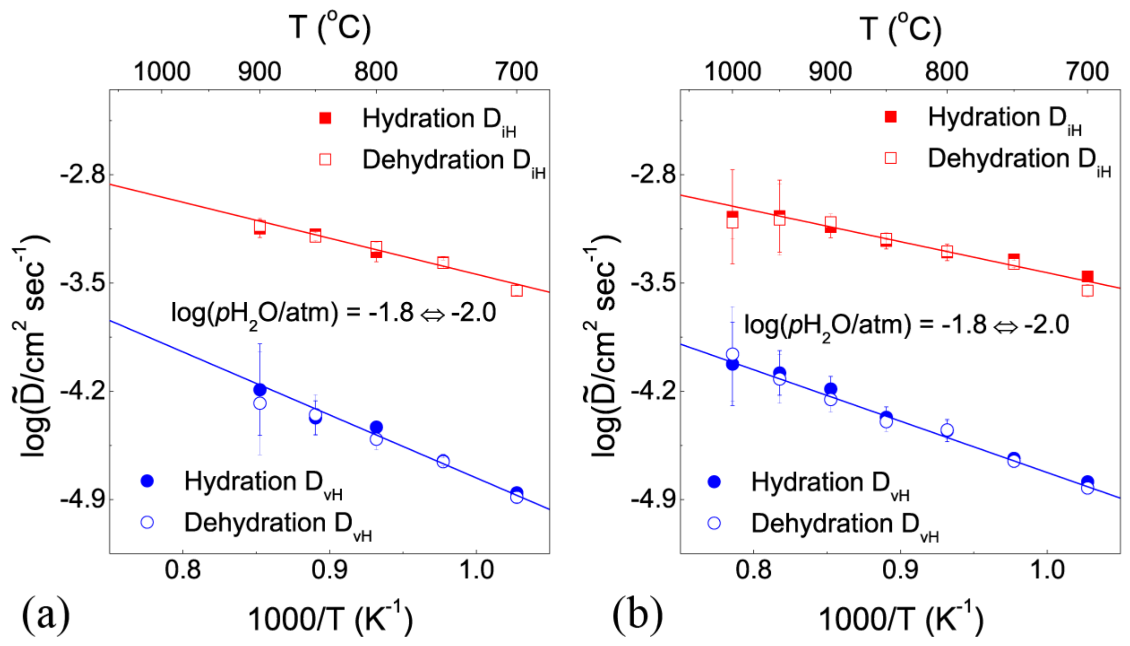

Supplementary Figure S5. Activation energies of chemical diffusivities from (a) Two-fold conductivity relaxation and (b) TG mass relaxation.
